# GLP2-GLP2R signal affects the viability and EGFR-TKIs sensitivity of PC9 and HCC827 cells

**DOI:** 10.1186/s12890-021-01800-3

**Published:** 2022-01-13

**Authors:** Bin Song, Hong Ge, Chenwei Pu, Ning Li

**Affiliations:** Department of Respiratory Medicine, Zibo Central Hospital, #54 Gongqingtuan West Road, Zhangdian, Zibo, 255036 Shandong People’s Republic of China

**Keywords:** EGFR-TKIs therapy, Lung cancer, GLP2R, Cisplatin

## Abstract

**Background:**

The resistance to epidermal growth factor receptor (EGFR)- tyrosine kinase inhibitors (TKIs) therapy is currently the major clinical challenge in the treatment of lung cancer. This study aims to reveal the role of glucagon-like peptide (GLP) 2 and GLP-2 receptor (GLP2R) signaling on the EGFR-TKIs and cisplatin resistance of lung cancer cells.

**Methods:**

The common differentially expressed genes in PC9 and HCC827 cells that were individually resistant to one of the three EGFR-TKIs (dacomitinib, osimertinib and afatinib) were screened. The data were from GSE168043 and GSE163913. The expression of GLP2R in drug-resistant cells was detected by western blot. The effect of GLP2R expression down- or up-regulation on resistance to dacomitinib, osimertinib, afatinib or cisplatin was measured by CCK-8 and flow cytometry assays. The long-acting analog of GLP-2, teduglutide, treated the parental cells.

**Results:**

A total of 143 common differentially expressed genes were identified. Compared with the parent cells, the GLP2R expression in drug-resistant cell lines was significantly up-regulated. The exogenous expression of GLP2R in parental cells enhanced cell viability, while knockdown of GLP2R levels in drug-resistant cell lines inhibited cell viability. In addition, teduglutide treatment also enhanced the viability of lung cancer cells.

**Conclusion:**

GLP2-GLP2R signal may change the sensitivity of cells to EGFR-TKIs and cisplatin. The development of GLP-2 or GLP2R inhibitors may be beneficial to the clinical treatment of lung cancer.

**Supplementary Information:**

The online version contains supplementary material available at 10.1186/s12890-021-01800-3.

## Background

Lung cancer remains the leading cause of cancer-related deaths worldwide, with more than 85% of lung cancer being non-small cell lung cancers (NSCLC) [1]. More than 70% of lung cancer patients have lymph node or distant metastasis [2]. Standard platinum-based chemotherapy is the cornerstone of systemic treatment for these patients, but it has only a modest effect on controlling tumor cell proliferation and improving the overall survival rate of patients [2, 3].

In the most recent decade, the individualized diagnosis and treatment of NSCLC has made great progress. Therapies targeting specific oncogenic driver gene mutations can inhibit the progression of NSCLC [4]. Among them, activating mutation of epidermal growth factor receptor (EGFR) drives the progression of NSCLC, and tumors with EGFR activating mutations (such as exon 19 deletion or exon 21 L858A mutation) are particularly sensitive to the treatment of EGFR tyrosine kinase inhibitors (TKIs) [5]. The standard first-line treatment of EGFR-TKIs includes first-generation (gefitinib, erlotinib), second-generation (afatinib) and third-generation (osimertinib) [6, 7]. EGFR-TKIs treatment improved the tumor response and progression-free survival time of patients [7]. Unfortunately, the median progression-free survival of patients with EGFR-mutant lung cancer treated with TKI is 8 to 13 months [7]. Almost all patients will eventually develop resistance and disease progression [8]. The resistance to EGFR-TKIs targeted therapy is currently a major clinical challenge in the treatment of lung cancer. Different mechanisms of acquired resistance to EGFR-TKIs have been reported [9]. Understanding and elucidating the molecular biology of EGFR mutant NSCLC resistance mechanisms can guide future drug development and more precise treatment progress.

The peptide hormones of the glucagon-like peptide (GLP) family are playing an increasingly important role in the clinical aspects of human diseases. For example, GLP-1 receptor-targeted imaging of insulinomas [10] and novel diabetes drugs based on GLP-1 [11]. Known nutritional and anti-inflammatory effects are also translated into the use of GLP-2 analogs to treat short bowel syndrome, Crohn’s disease and Inflammatory bowel disease [12, 13]. The GLP-2 receptor (GLP2R) mediates the actions of GLP-2, which belongs to the G protein-coupled receptor superfamily and is a member of the glucagon receptor family [14].

In this study, the role of GLP2-GLP2R signal on EGFR-TKIs and cisplatin resistance of NSCLC cells was performed.

## Methods

### Differentially expressed genes screen

The original data of GSE168043 (Parent cells VS. Dacomitinib resistant PC9 cells) and GSE163913 (Parent cells VS. Osimertinib or afatinib resistant PC9 or HCC827 cells) were downloaded from the GEO database. Differentially expressed genes were screened using *P* < 0.01 and log_2_ (FC) > 1 or < -1 as criteria, and enriched with GO function using Funrich 3.0 software. The GLP2R 2D structure was from STRING [15].

### Cell culture

Human lung adenocarcinoma PC9 and HCC827 cell lines (harboring EGFR exon 19 deletion), A549 and A549/DDP cell lines were obtained from the Cell Bank of Type Culture Collection of the Chinese Academy of Sciences, and cultured with DMEM containing 10% FBS (Hyclone, GE Healthcare) and 1% penicillin–streptomycin (Gibco, Thermo Fisher Scientific) with an atmosphere of 5% CO_2_ at 37˚C. A549/DDP cell line was resistant to 2 μg/ml DDP. PC9 or HCC827 cells were treated with increasing concentrations of dacomitinib (PC-DR or HCC287-DR), osimertinib (PC-OR or HCC287-OR) or afatinib (PC-AR or HCC287-AR) (from 1 pM to 100 nM) for 6 months to obtain drug-resistant cell lines [16].

### Transfection and reagent

siRNA targeting GLP2R mRNA to down-regulate GLP2R expression (GLP2R-KD) was purchased from RiBoBio (Guangzhou, China). The GLP2R cDNA was amplified and cloned into pcDNA3.1 plasmid (GLP2R). The siRNA and overexpression plasmid were transfected into cells with Lipofectamine 2000 reagent. Dacomitinib (CAS No. 1110813-31-4, S2727), osimertinib (CAS No. 1421373-65-0, S7297), afatinib (CAS No. 850140-72-6, S1011) and cisplatin (CAS No. 15663-27-1, S1166) were obtained from Selleck.cn. Teduglutide (M10249) was purchased from AbMole.

### Western blot

The cells were homogenized using ice-cold RIPA strong lysis buffer (Beyotime Institute of Biotechnology), and total protein was collected by centrifugation at 10,000*g* for 10 min at 4˚C. The protein concentration was determined using a bicinchoninic acid assay. 10 µg of protein was subjected to SDS-PAGE electrophoresis, then transferred to a PVDF membrane (Bio-Rad), and blocked with 5% skimmed milk for 2 h at room temperature. The membrane was probed with anti-GLP2R (Rabbit Polyclonal, 1:2000, PA5-104301, Thermo Fisher Scientific). Subsequently, the membrane was incubated with a horseradish peroxidase-conjugated goat anti-rabbit antibody (1:5000, ab6721, Abcam) at room temperature for 1.5 h. Signals were visualized with enhanced chemiluminescence solution (EMd Millipore) and developed using the chemiluminescence apparatus (GE Healthcare). Optical density analysis was performed using Quantity One 4.6.7 (BioRad Laboratories).

### CCK-8 assay

The CCK-8 experiment was used to detect cell proliferation. The cells were seeded into a 96-well plate at a density of 1 × 10^4^ cells/well and subjected to single drug treatment (100 nm dacomitinib, osimertinib or afatinib, or 10 μg/ml cisplatin) for 24 and 48 h. The old medium was removed, and the cells were incubated with 10% CCK-8 reagent for 2 h at 37˚C. Absorbances of each well at 450 nm were measured using a microplate reader (iMARK, Bio-Rad Laboratories).

### Flow cytometry assay

The cells which subjected to single drug treatment (100 nm dacomitinib, osimertinib or afatinib, or 10 μg/ml cisplatin) for 48 h were collected and stained using an Annexin V-FITC/PI kit (cat. no. cA1020, Beijing Solarbio Science & Technology Co., Ltd.) according to the manufacturer's instructions. The proportion of apoptotic cells was analyzed using flow cytometry (Bd Biosciences) and FlowJo 7.6 software (Becton, Dickinson and Company).

### Statistical analysis

Data was analyzed with SPSS 21.0 and GraphPad Prism 6.0, and presented as the mean ± standard deviation. Differences between multiple groups were compared using one-way ANOVA with a post-hoc Dunnett's or Bonferroni test. Differences between two groups were analyzed using Student's t-test. *P* < 0.05 was considered to be statistically significant.

## Results

### GLP2R is differentially expressed in PC and HCC827 cells resistant to EGFR-TKIs

**Fig. 1:**
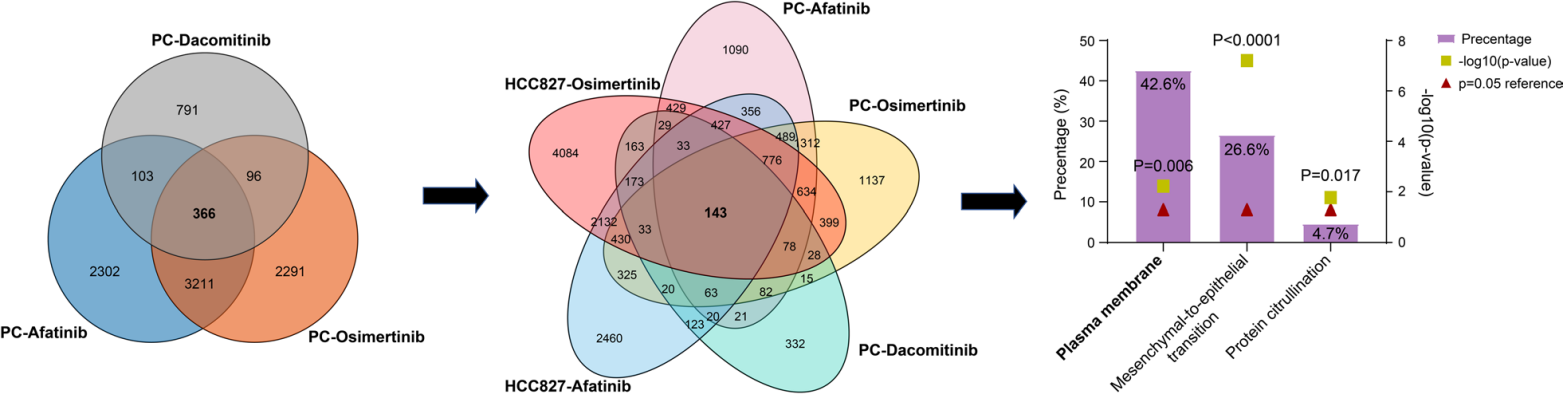
Screening of differentially expressed genes in PC and HCC827 cells resistant to EGFR-TKIs. Left: the differentially expressed genes in PC9 cells co-resistant to the three EGFR-TKIs (Dacomitinib, Osimertinib and Afatinib) were screened using *P* < 0.01 and log_2_ (FC) > 1 or < − 1 as criteria (n = 366). The data from GSE168043 and GSE163913. Middle: the differentially expressed genes of PC and HCC827 cells co-resistant to EGFR-TKIs (n = 143). Right: 143 differentially expressed genes were enriched with GO function using Funrich 3.0 software

**Fig. 2:**
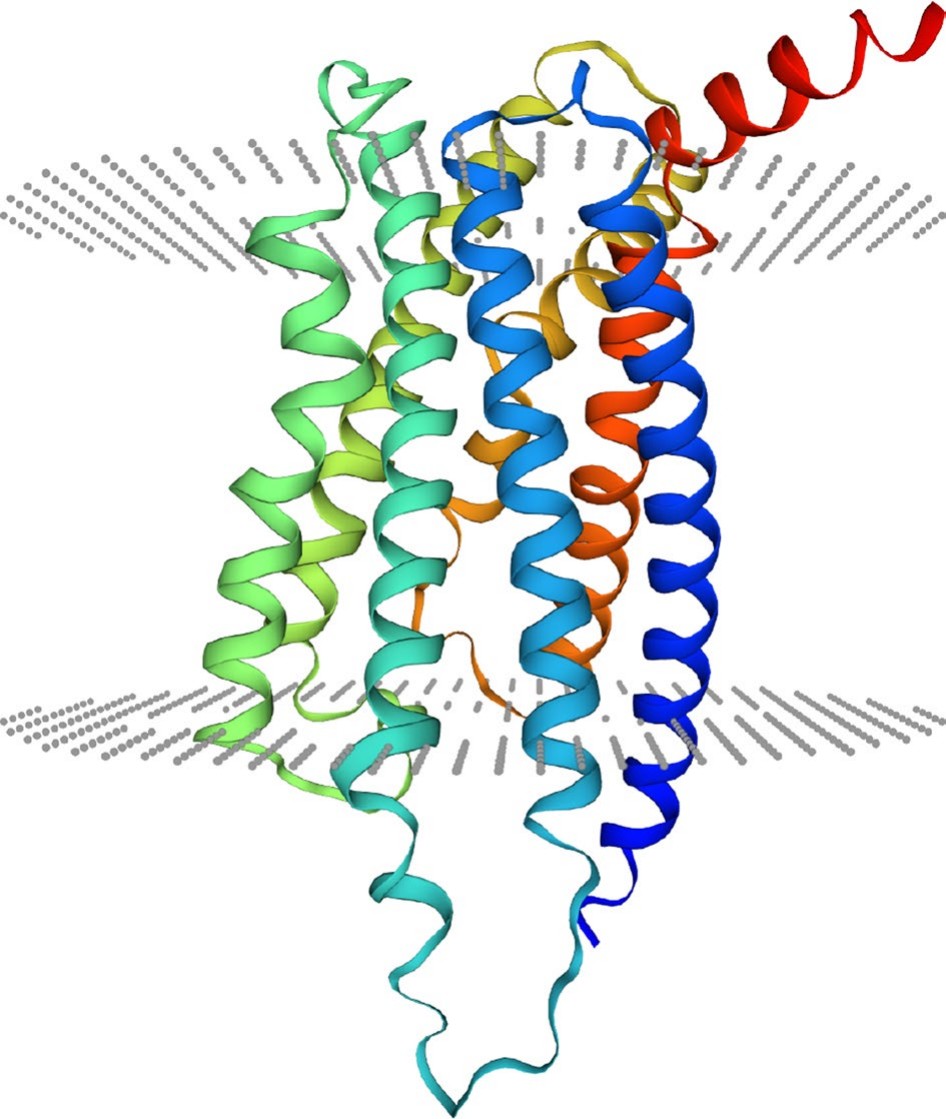
GLP2R 2D structure. The data from STRING

The common differentially expressed genes in PC9 cells that were individually resistant to one of the three EGFR-TKIs (Dacomitinib, Osimertinib and Afatinib) were screened (n = 366, Fig. 1). Combined with the transcriptome information of HCC827 cells resistant to osimertinib or afatinib, 143 common differentially expressed genes were identified (Fig. 1). Subsequently, 143 differentially expressed genes were enriched for GO functions. The biological pathways (mesenchymal-epithelial transformation, MET and protein citrullination) and cellular components (plasma membrane) were screened out (n = 49, *p* < 0.05, Fig. 1). Since chemotherapy resistance is closely related to transmembrane proteins, we finally selected GLP2R from the plasma membrane components as the target of this study (Fig. 2).

### GLP2-GLP2R signal affects the viability and EGFR-TKIs sensitivity of PC9 and HCC827 cells

**Fig. 3:**
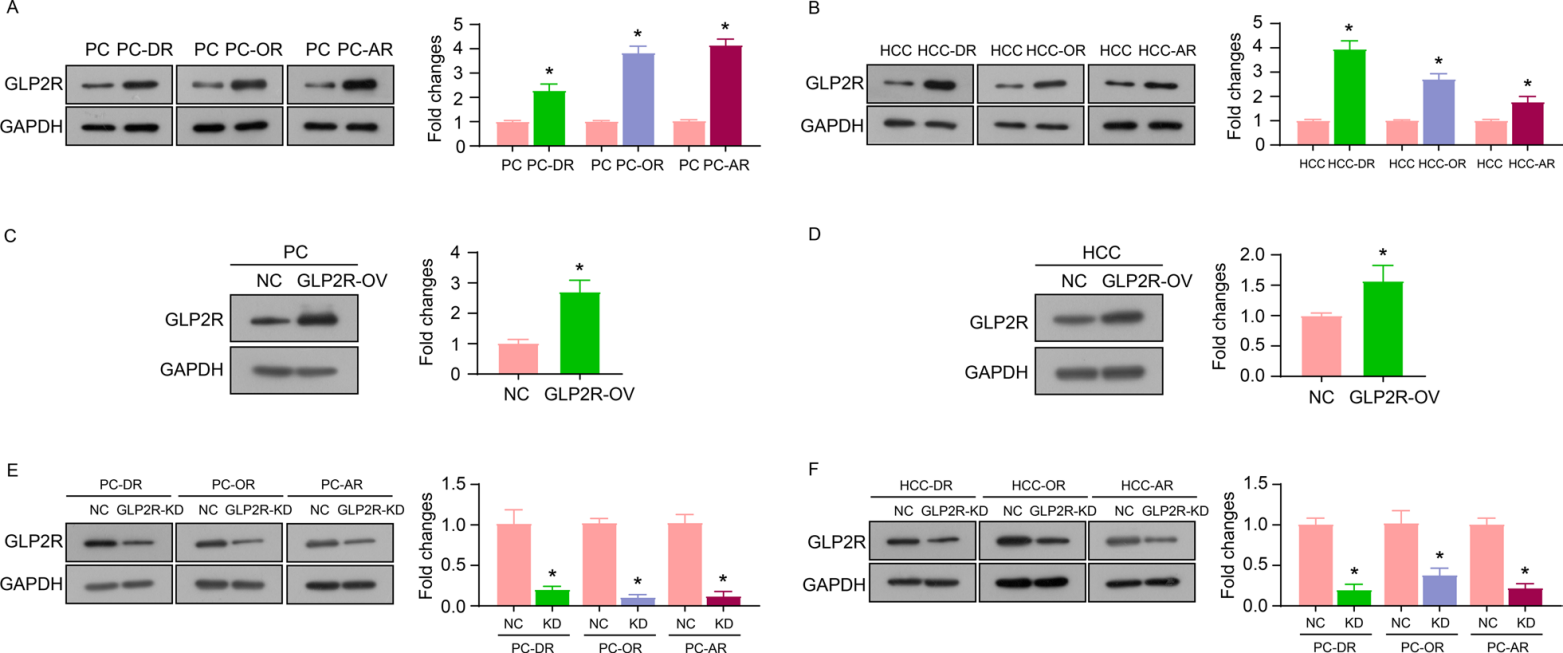
GLP2R expression. The protein expression of in parent or drug-resistant PC9 (**A**) and HCC827 (**B**) cells. **C**, **D** GLP2R expression in parental cells was up-regulated by overexpression plasmid transfection (PC-GLP2R-OV or HCC287-GLP2R-OV). **E**, **F** GLP2R expression in drug-resistant cell lines was down-regulated by siRNA transfection (PC-XR + GLP2R-KD or HCC287-XR + GLP2R-KD). The parent cells, PC or HCC287; parental cells exogenously express GLP2R, GLP2R-OV; PC9 or HCC827 cells resistant to dacomitinib (PC-DR or HCC287-DR), osimertinib (PC-OR or HCC287-OR) or afatinib (PC-AR or HCC287-AR); knockdown of GLP2R levels in drug-resistant cell, GLP2R-KD. *P < 0.05

**Fig. 4:**
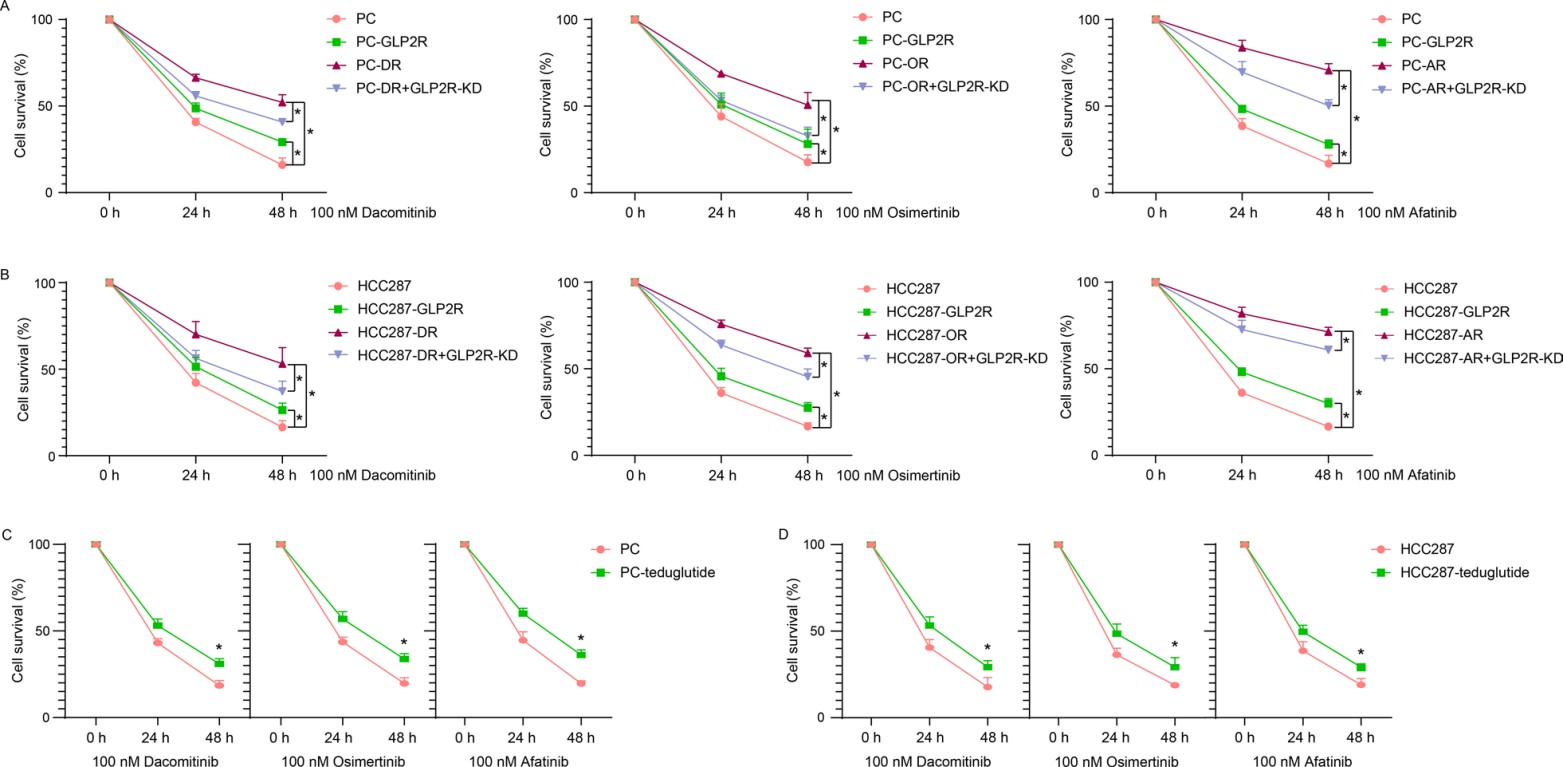
GLP2-GLP2R signal affects the sensitivity of PC9 and HCC827 cells to EGFR-TKIs. The cell survival of parent or drug- resistant PC9 (**A**) and HCC827 (**B**) cells transfected with pcDNA3.1-GLP2R plasmid or siRNA-GLP2R. The cell survival of PC9 (**C**) and HCC827 (**D**) cells treated with teduglutide (0.5 μM). The parent cells, PC or HCC287; parental cells exogenously express GLP2R, PC-GLP2R or HCC287-GLP2R; PC9 or HCC827 cells resistant to dacomitinib (PC-DR or HCC287-DR), osimertinib (PC-OR or HCC287-OR) or afatinib (PC-AR or HCC287-AR); knockdown of GLP2R levels in drug-resistant cell, PC-XR + GLP2R-KD or HCC287-XR + GLP2R-KD. *P < 0.05

Subsequently, PC9 and HCC827 cells resistant to dacomitinib (PC-DR or HCC287-DR), osimertinib (PC-OR or HCC287-OR) or afatinib (PC-AR or HCC287-AR) were identified, and the expression of GLP2R protein in these cells was detected. As shown in Fig. 3A and B, compared with the parent cells (PC or HCC287), the protein expression of GLP2R in drug-resistant cell lines was significantly up-regulated. Therefore, the expression of GLP2R in parental cells was up-regulated by overexpression plasmid transfection (PC-GLP2R-OV or HCC287-GLP2R-OV) (Fig. 3C and D), and the expression of GLP2R in drug-resistant cell lines was down-regulated by siRNA transfection (PC-XR + GLP2R-KD or HCC287-XR + GLP2R-KD) (Fig. 3E and F), and the effect of GLP2R expression on cell viability was detected using CCK-8 experiment. As shown in Fig. 4A and B, the exogenous expression of GLP2R (PC-GLP2R or HCC287-GLP2R) in parental cells (PC or HCC287) enhanced cell viability, while the knockdown of GLP2R levels in drug-resistant cell lines (PC-XR + GLP2R-KD or HCC287-XR + GLP2R-KD) inhibited cell viability. In addition, we also treated the parental cells with teduglutide (0.5 μM), a long-acting analog of GLP-2, and found that teduglutide treatment also enhanced the viability of PC9 and HCC827 cells (Fig. 4C and D). These results indicated that GLP2-GLP2R signal affects the viability of PC9 and HCC827 cells, as well as the sensitivity to dacomitinib, osimertinib and afatinib.

### GLP2-GLP2R signal is involved in the viability and cisplatin resistance of A549 cells

**Fig. 5:**
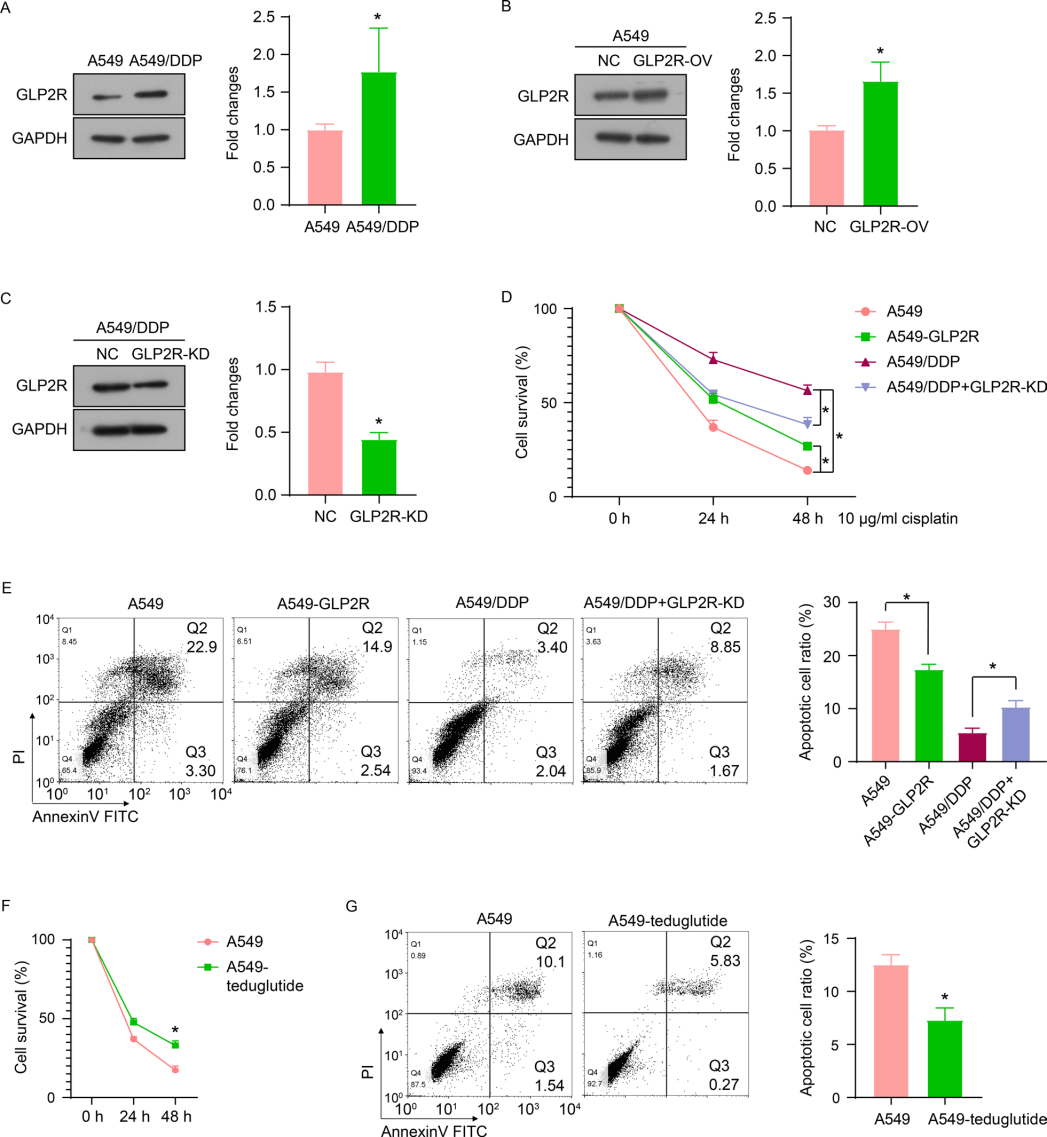
GLP2-GLP2R signal is involved in the cisplatin resistance mechanism of A549 cells. **A** The protein expression of GLP2R in A549 and A549/DDP cells. GLP2R expression in A549 cells was up-regulated by overexpression plasmid transfection (**B**), in A549/DDP cells was down-regulated by siRNA transfection (**C**). The cell survival (**D**) and apoptosis (**E**) of A549 and A549/DDP cells transfected with pcDNA3.1-GLP2R plasmid or siRNA-GLP2R. The cell survival (**F**) and apoptosis (**G**) of A549 and A549/DDP cells treated with teduglutide (0.5 μM). *P < 0.05

Finally, we tested the effect of GLP2R expression on the cisplatin resistance of A549 cells. Compared with A549 cells, the expression of GLP2R in the A549/DDP cell line was significantly up-regulated (Fig. 5A). Similarly, the expression of GLP2R in A549 cells was up-regulated by overexpression plasmid transfection (Fig. 5B), and the expression of GLP2R in A549/DDP cells was down-regulated by siRNA transfection (Fig. 5C). GLP2R overexpression significantly enhanced the viability of A549 cells and inhibited apoptosis, while GLP2R knockdown inhibited the viability of A549/DDP cells and induced apoptosis (Fig. 5D and E). In addition, teduglutide treatment (0.5 μM) enhanced the viability of A549 cells and inhibited apoptosis (Fig. 5F and G). These results indicated that GLP2-GLP2R signal may be involved in the viability and cisplatin resistance of A549 cells.

## Discussion

When most patients are diagnosed with NSCLC, they lose the opportunity for surgery or radical resection. The only treatment is chemotherapy which only prolongs short-term survival time and has serious side effects [17]. When the first generation of EGFR-TKIs came out, NSCLC patients with EGFR gene activating mutations have a new treatment method. Compared with patients receiving platinum-based chemotherapy, this method can prolong progression-free survival (PFS) and improve life quality [18]. However, patients treated with EGFR-TKIs soon inevitably develop resistance and disease deterioration [19]. The mechanism of EGFR-TKIs resistance is still unclear. Some mechanisms have been reported, including the acquisition of the T790M gatekeeper mutation [20], kinase switching caused by the mesenchymal-epithelial transition (MET) amplification [21] and PIK3CA mutation [22]. The development of new treatment strategies to overcome EGFR TKI resistance is an urgent clinical goal.

This study analyzed the RNA-Seq results of PC9 and HCC827 cells resistant to dacomitinib, osimertinib and afatinib, and obtained 143 differentially expressed genes. These genes were mainly GO enriched into cellular components (plasma membrane, n = 49). At the cellular level, the uptake or outflow of drugs mediated by membrane transporters makes target cells have drug-sensitive or drug-resistant phenotypes, thereby affecting the therapeutic effect [23]. Transporters have become a key determinant of drug disposal, efficacy and adverse drug reactions [24]. In this study, the role of GLP2R on the resistance of NSCLC cells to EGFR-TKIs and cisplatin was performed.

We found that GLP2R was highly expressed in NSCLC cells resistant to dacomitinib, osimertinib, afatinib or cisplatin. The expression of GLP2R may change the cell sensitivity to drugs. In addition, the long-acting analog of GLP-2, teduglutide also affected the resistance of NSCLC cells. These results indicated that GLP2-GLP2R signal may be involved in the EGFR-TKIs and cisplatin resistance mechanism of NSCLC. Furthermore, the development of GLP-2 or GLP2R inhibitors may be benefit the clinical treatment of NSCLC.

However, only a few studies using immunohistochemical methods have reported the expression of GLP2R in a limited number of tumor types, including ileal carcinoid [25], gastrointestinal stromal tumors [26] and cervical cancer [27]. In addition, studies have reported that GLP-2 and its analogs stimulate the proliferation, migration and invasion of myofibroblasts and cancer cells through the IGF pathway [28, 29]. In various rodent models of colorectal cancer, GLP-2 administration led to an increase in the number of colorectal cancer precursor lesions (30, 31). Although, little is known about the role of GLP2-GLP2R signaling in tumors. But these results all indicate the important role of GLP2-GLP2R signaling in tumor progression and drug resistance (Additional files 1, 2, 3, 4, 5, 6, 7).

## Conclusion

In conclusion, GLP2R was highly expressed in NSCLC cells resistant to EGFR-TKIs or cisplatin. The GLP2-GLP2R signal may change the sensitivity of cells to drugs, and this observation would explain that GLP2-GLP2R signal may one of the players to establish drug resistance of lung cancer cells. The development of GLP-2 or GLP2R inhibitors may be beneficial to the clinical treatment of NSCLC.

## Supplementary Information

**Additional file 1.** The original blots of Figure 3A.

**Additional file 2.** The original blots of Figure 3B.

**Additional file 3.** The original blots of Figure 5A.

**Additional file 4.** The original blots of Figure 5C.

**Additional file 5.** The original blots of Figures 3C–D and 5B.

**Additional file 6.** The original blots of Figure 3E.

**Additional file 7.** The original blots of Figure 3F.

## Data Availability

The datasets used and/or analysed during the current study are available from the corresponding author on reasonable request.
